# Supplementary material for: Lung Cancer Screening in Greece: A Modelling Study to Estimate the Impact on Lung Cancer Life Years
Source: Cancers (Basel). 2022 Nov 8;14(22):5484. doi: 10.3390/cancers14225484 (PMC9688856; doi:10.3390/cancers14225484)
Supplement: Supplementary file 1 [file cancers-14-05484-s001.zip › cancers-1963561-supplementary.pdf]

Table S1. Health state distribution

Health state distribution		Source
Healthy	0.99612	Own calculation based on Globocan 2020 and Greek statistics on smoking population
Patient	0.00388	
NSCLC- Stage 0	1.00%	Clinical expert opinion
NSCLC- Stage I	6.62%	Clinical expert opinion
NSCLC- Stage II	10.86%	Clinical expert opinion
NSCLC- Stage III operable	11.46%	Clinical expert opinion
NSCLC- Stage III inoperable	20.47%	Clinical expert opinion
NSCLC- Stage IV	33.59%	Clinical expert opinion
SCLC – limited stage	5.42%	Clinical expert opinion
SCLC – extensive stage	10.58%	Clinical expert opinion

Table S2. Probability of random cancer-unrelated screening

Total CTs in Greece	2,295,689	Eurostat (online data codes: hlth_co_exam and hlth_rs_equip)
Chest CTs % of total	17.00%	Papadimitriou et al., 2003
LC patients CTs per year (average)	4	Clinical expert opinion
LC patients	9,300	Clinical expert opinion
Cancer-unrelated CT probability	3.84%	Own calculation (based on Greek population aged 15+, ELSTAT data)

Table S3. Probability of developing symptoms by cancer type and stage

Symptoms	NSCLC				SCLC	
	stage I	stage II	stage III	stage IV	Limited	Extensive
**No**	0.593	0.425	0.302	0.277	0.174	0.109
**Yes**	0.407	0.575	0.698	0.723	0.826	0.891

Source: Lung cancer symptoms at diagnosis: results of a nationwide registry study

Table S4. Mean progression time per cancer type and stage

Mean progression time (treated)	Months	Source
From NSCLC Stage I to Stage IIIA	24.80	Clinical expert opinion

From NSCLC Stage II to Stage IIIA	16.80	Clinical expert opinion
From NSCLC Stage IIIA to Stage IV	11.67	Clinical expert opinion
From NSCLC Stage IIIB to Stage IV	7.50	Clinical expert opinion
From SCLC limited to extensive	9.00	Clinical expert opinion
**Mean progression time (untreated)**		
From NSCLC Stage I to Stage IIIA	6.43	Clinical expert opinion
From NSCLC Stage II to Stage IIIA	5.60	Clinical expert opinion
From NSCLC Stage IIIA to Stage IV	6.00	Clinical expert opinion
From NSCLC Stage IIIB to Stage IV	2.57	Clinical expert opinion
From SCLC limited to extensive	2.43	Clinical expert opinion

Table S5. Transition probabilities for NSCLC stage I

NSCLC- Stage I, 3-month interval		
**State**	**Prob.**	**Source**
Treated patients (remain alive)	97.42%	Clinical expert opinion
Treated patients (die)	0.30%	Clinical expert opinion
Treated patients (Progress to Stage III)	1.49%	Clinical expert opinion
Treated patients (Progress to Stage IV)	0.79%	Clinical expert opinion
Untreated patients (remain alive)	74.83%	Clinical expert opinion
Untreated patients (die)	1.00%	Clinical expert opinion
Untreated patients (Progress to Stage III)	18.14%	Clinical expert opinion
Untreated patients (Progress to Stage IV)	6.03%	Clinical expert opinion

Table S6. Transition probabilities for NSCLC stage II

NSCLC- Stage II, 3-month interval		
**State**	**Prob.**	**Source**
Treated patients (remain alive)	87.67%	Clinical expert opinion
Treated patients (die)	2.50%	Clinical expert opinion
Treated patients (Progress to Stage III)	4.11%	Clinical expert opinion
Treated patients (Progress to Stage IV)	5.72%	Clinical expert opinion
Untreated patients (remain alive)	62.87%	Clinical expert opinion
Untreated patients (die)	2.56%	Clinical expert opinion

Untreated patients (Progress to Stage III)	22.92%	Clinical expert opinion
Untreated patients (Progress to Stage IV)	11.65%	Clinical expert opinion

Table S7. Transition probabilities for NSCLC stage IIIA

**NSCLC- Stage IIIA, 3-month interval**		
**State**	**Prob.**	**Source**
Treated patients (remain alive)	62.84%	Clinical expert opinion
Treated patients (die)	4.90%	Clinical expert opinion
Treated patients (Progress to Stage IV)	12.23%	Clinical expert opinion
Treated patients (Downstage to Stage II)	20.03%	Clinical expert opinion
Untreated patients (remain alive)	49.10%	Clinical expert opinion
Untreated patients (die)	12.50%	Clinical expert opinion
Untreated patients (Progress to Stage IV)	37.20%	Clinical expert opinion
Untreated patients (Downstage to Stage II)	1.20%	Clinical expert opinion

Table S8. Transition probabilities for NSCLC stage IIIB

**NSCLC- Stage IIIB, 3-month interval**		
**State**	**Prob.**	**Source**
Treated patients (remain alive)	56.8%	Clinical expert opinion
Treated patients (die)	8.80%	Clinical expert opinion
Treated patients (Progress to Stage IV)	17.30%	Clinical expert opinion
Treated patients (Downstage to Stage II)	17.20%	Clinical expert opinion
Untreated patients (remain alive)	34.30%	Clinical expert opinion
Untreated patients (die)	24.50%	Clinical expert opinion
Untreated patients (Progress to Stage IV)	41.20%	Clinical expert opinion
Untreated patients (Downstage to Stage II)	0.00%	Clinical expert opinion

Table S9. Transition probabilities for NSCLC stage IV

**NSCLC- Stage IV, 3-month interval**		
**State**	**Prob.**	**Source**
Treated patients (remain alive)	83.50%	Clinical expert opinion

Treated patients (die)	16.50%	Clinical expert opinion
Untreated patients (remain alive)	56.00%	Clinical expert opinion
Untreated patients (die)	44.00%	Clinical expert opinion

Table S10. Transition probabilities for SCLC limited stage

**SCLC-limited, 3-month interval**		
**State**	**Prob.**	**Source**
Treated patients (remain alive)	76.42%	Clinical expert opinion
Treated patients (die)	8.20%	Clinical expert opinion
Treated patients (Progress to Extensive Stage)	15.39%	Clinical expert opinion
Untreated patients (remain alive)	37.70%	Clinical expert opinion
Untreated patients (die)	33.50%	Clinical expert opinion
Untreated patients (Progress to Extensive Stage)	28.80%	Clinical expert opinion

Table S11. Transition probabilities for SCLC extensive stage

**SCLC- extensive, 3-month interval**		
**State**	**Prob.**	**Source**
Treated patients (remain alive)	82.00%	Clinical expert opinion
Treated patients (die)	18.00%	Clinical expert opinion
Untreated patients (remain alive)	28.00%	Clinical expert opinion
Untreated patients (die)	72.00%	Clinical expert opinion

Table S12. Health state distribution in the hypothetical scenario (100% screening)

**Health state distribution**		**Source**
Healthy	0.99612	Own calculation based on Globocan 2020 and Greek statistics on smoking population
Patient	0.00388	
NSCLC- Stage 0	2.80%	Clinical expert opinion
NSCLC- Stage I	16.20%	Clinical expert opinion
NSCLC- Stage II	18.50%	Clinical expert opinion

NSCLC- Stage III operable	15.70%	Clinical expert opinion
NSCLC- Stage III inoperable	15.50%	Clinical expert opinion
NSCLC- Stage IV	18.20%	Clinical expert opinion
SCLC – limited stage	4.90%	Clinical expert opinion
SCLC – extensive stage	8.20%	Clinical expert opinion
